# Upregulation of Peroxiredoxin 3 Protects *Afg3l*2-KO Cortical Neurons *In Vitro* from Oxidative Stress: A Paradigm for Neuronal Cell Survival under Neurodegenerative Conditions

**DOI:** 10.1155/2019/4721950

**Published:** 2019-10-31

**Authors:** Barbara Bettegazzi, Ilaria Pelizzoni, Floramarida Salerno Scarzella, Lisa Michelle Restelli, Daniele Zacchetti, Francesca Maltecca, Giorgio Casari, Fabio Grohovaz, Franca Codazzi

**Affiliations:** ^1^Università Vita-Salute San Raffaele, Via Olgettina 58, 20132 Milan, Italy; ^2^Ospedale San Raffaele, Via Olgettina 60, 20132 Milan, Italy; ^3^Telethon Institute of Genetics and Medicine (TIGEM), Naples, Italy

## Abstract

Several neurodegenerative disorders exhibit selective vulnerability, with subsets of neurons more affected than others, possibly because of the high expression of an altered gene or the presence of particular features that make them more susceptible to insults. On the other hand, resilient neurons may display the ability to develop antioxidant defenses, particularly in diseases of mitochondrial origin, where oxidative stress might contribute to the neurodegenerative process. In this work, we investigated the oxidative stress response of embryonic fibroblasts and cortical neurons obtained from *Afg3l2*-KO mice. *AFG3L2* encodes a subunit of a protease complex that is expressed in mitochondria and acts as both quality control and regulatory enzyme affecting respiration and mitochondrial dynamics. When cells were subjected to an acute oxidative stress protocol, the survival of AFG3L2-KO MEFs was not significantly influenced and was comparable to that of WT; however, the basal level of the antioxidant molecule glutathione was higher. Indeed, glutathione depletion strongly affected the viability of KO, but not of WT MEF, thereby indicating that oxidative stress is more elevated in KO MEF even though well controlled by glutathione. On the other hand, when cortical KO neurons were put in culture, they immediately appeared more vulnerable than WT to the acute oxidative stress condition, but after few days in vitro, the situation was reversed with KO neurons being more resistant than WT to acute stress. This compensatory, protective competence was not due to the upregulation of glutathione, rather of two mitochondrial antioxidant proteins: superoxide dismutase 2 and, at an even higher level, peroxiredoxin 3. This body of evidence sheds light on the capability of neurons to activate neuroprotective pathways and points the attention to peroxiredoxin 3, an antioxidant enzyme that might be critical for neuronal survival also in other disorders affecting mitochondria.

## 1. Introduction

*AFG3L2* (ATPase family gene 3-like 2) encodes a subunit of the large m-AAA (ATPases associated with various cellular activities) protease complex expressed on the inner membrane of mitochondria and active on the matrix side. In humans, AFG3L2 is either part of a homohexameric complex or associated in a heterocomplex with the homologous protein paraplegin (encoded by *SPG7*), whose mutations are responsible for hereditary spastic paraplegia. AFG3L2 is crucial for several mitochondrial functions: it is reported to contribute to the quality control system, to play chaperon-like activities, to regulate the processing of proteins involved in mitochondrial dynamics, and to control the turnover of respiratory chain subunits [1–5]. Heterozygous missense or frameshift mutations [3, 6] as well as partial deletion [7] of *AFG3L2* have been associated to spinocerebellar ataxia type 28 (SCA28), characterized by autosomal dominant inheritance [8] (https://www.omim.org). SCA28 [9] is characterized by young-adult onset, with cerebellar atrophy but no signs of cognitive impairment and sensory involvement [10]. On the other hand, a homozygous missense mutation in AFG3L2 causes the spastic ataxia type 5 (SPAX5), a different and more severe disease, characterized by early-onset spasticity, myoclonic epilepsy, cerebellar atrophy, oculomotor apraxia, and dystonia [11]. Recently, also early-onset optic atrophy has been associated with a *de novo* AFG3L2 heterozygous mutation (p.R468C; [12–14]). The *Afg3l2* haploinsufficiency in SCA28 causes several mitochondrial dysfunctions that include reduced assembly of respiratory complexes, swollen appearance, fragmentation and altered dendritic distribution, increase in oxidative stress, and calcium dysregulation [15, 16]. The effects of mutations are particularly evident in Purkinje cells (PCs), where AFG3L2 is highly expressed [3, 17] and where the reduced capability in buffering calcium by the affected mitochondria was proposed to cause the so-called “dark cell degeneration” [18]. *Afg3l2* missense mutation or haploinsufficiency was also reported to induce an increase in lipid peroxidation in lymphoblastoid cell lines [19] and protein oxidation in mouse cerebellum from KO and heterozygous mice [18, 20], respectively. Indeed, although haploinsufficiency does not mimic the genetic background of patients, it should be considered that the mutations in the proteolytic domain of Afg3l2, while not altering the protein levels, still reduce the overall activity of the complex of about 50%, therefore providing a functional haploinsufficiency [5]. Overall, the heterozygous models functionally recapitulate the genetic settings of SCA28 patients and are currently used to study the molecular alterations in this disease.

Higher levels of oxidative stress play a relevant role not only in SCA28 but also in other neurodegenerative disorders, especially those with mitochondrial origin (such as Friedreich's ataxia or hereditary spastic paraplegia type 7 (HSP-SPG7)), or severely affecting these organelles (such as amyotrophic lateral sclerosis). It is widely accepted that alterations of mitochondria, which represent the main site of reactive oxygen species (ROS) production, increase the already high level of oxidative stress in neuronal cells due to high oxygen consumption, autooxidation of neurotransmitters, elevations of intracellular Ca^2+^ concentration during synaptic activity, and age-dependent increase in iron and accompanied by low expression of antioxidant defenses [21, 22]. Among them, peroxiredoxins (Prxs) seem to play a crucial role in detoxifying hydrogen peroxide [23] that, in turn, is converted into the more reactive hydroxyl radicals.

Prxs represent a family of thiol peroxidases ubiquitously and abundantly expressed in mammalian cells, which is extremely efficient in oxidant perception and fast in scavenging activity [23]. There are six mammalian Prxs, classified in three subtypes (typical 2-Cys, atypical 2-Cys, and typical 1-Cys) depending on their catalytic mechanism of peroxide reduction [24, 25]. Their high reactivity and specificity toward hydrogen peroxide and their inactivation by hyperoxidation, when the peroxides are in excess, open the possibility that Prxs may act as cellular redox sensors. This could be particularly true for Prx3, a typical 2-Cys member of the family, which is selectively located in the mitochondrial matrix, where most of cellular hydrogen peroxide is generated as a byproduct of aerobic respiration. Recent data indicate that Prx3 account for almost 85-90% of hydrogen peroxide detoxification, therefore defining a mitochondrial redox setpoint [26]. Hence, Prx3 might play a crucial role when mitochondrial oxidants are in excess; therefore, the capability of some neurons to significantly increase Prx3 expression might represent a fundamental protective mechanism counteracting the consequences of oxidative stress caused by local insults or due to genetic diseases.

In this study, we investigate the effects of oxidative stress conditions in murine embryonic fibroblasts (MEFs) and cortical neurons obtained from *AFG3L2*-KO and WT mice. We found that in both cell types, specific antioxidant pathways are induced by oxidative stress; in particular, Prx3 appears to be massively upregulated in cortical neurons where it might play a relevant protective role.

## 2. Material and Methods

### 2.1. Materials

Cell culture media and reagents were from Lonza (Basel, Switzerland), culture flasks and multiwell plates from Nalge Nunc (Rochester, NY, USA), and Petri dishes from Falcon BD (Franklin Lakes, NJ, USA). When not specified, fluorescent dyes were from Molecular Probes and Thermo Fisher Scientific, and chemicals were from Sigma-Aldrich (St. Louis, MO, USA).

### 2.2. MEF Lines

Primary MEFs were established from embryonic day E16.5 *Afg3l2^+/+^* (WT) and *Afg3l2^–/–^* (KO) mice embryos and immortalized by SV40, using 300 *μ*g/ml geneticin for selection, as already described [4]. MEFs were grown in DMEM supplemented with 5% fetal clone III, 2 mM glutamine, 100 *μ*g/ml Pen Strep, and 1 mM sodium pyruvate. Cells were maintained at 37°C in a humidified 5% CO_2_ atmosphere; 1 day before the experiments, cells were detached with trypsin and replated onto poly-lysine-coated glass coverslips or plastic multiwell plates.

### 2.3. Primary Cortical Neurons

Primary cultures of cortical neurons were prepared from newborn *Afg3l2^+/+^* and *Afg3l2^–/–^* mice. Briefly, after quick subdivision of cortices into small sections, the tissue was incubated in a dissection medium (Dulbecco's modified Eagle's medium supplemented with 4.5 g/l of glucose and 20 mM Hepes) containing 100 U of papain, 0.5 mg/ml DNase type IV (Calbiochem, La Jolla, CA, USA), and 0.5 mg/ml L-cysteine hydrochloride for 15 min at 34°C. The pieces were then mechanically dissociated in the dissection medium supplemented with 0.5 mg/ml DNase IV. After centrifugation, cells were plated onto poly-L-lysine-coated plates and maintained in minimal essential medium supplemented with 20 mM glucose, B27 (Life Technologies, Carlsbad, CA, USA), 2 mM glutamax, 5% fetal clone III (FCIII; Hyclone, South Logan, UT, USA), and 5 mM 1-b-D-cytosine-arabinofuranoside. Cultures were maintained at 37°C in a 5% CO_2_ humidified incubator and used between 2 and 8 days after plating.

### 2.4. Videomicroscopy Setup

The video imaging setup is based on an Axioskop 2 microscope (Zeiss, Oberkochen, Germany) and a Polychrome IV (Till Photonics, GmbH, Martinsried, Germany) light source. Fura-2 was excited at 360 nm (the calcium-insensitive wavelength) to monitor Fe^2+^ variations (as quenching of the fluorescence signal). Fluorescence images were collected by a cooled CCD video camera (PCO Computer Optics GmbH, Kelheim, Germany). The “Vision” software (Till Photonics) was used to control the acquisition protocol and to perform data analysis.

The Array Scan XTI platform (Thermo Fisher Scientific) was used for HTM.

### 2.5. Solutions and Dye Loading

Dye loading and single-cell experiments were performed in Krebs Ringer Hepes buffer (KRH, containing 5 mM KCl, 125 mM NaCl, 2 mM CaCl_2_, 1.2 mM MgSO_4_, 1.2 mM KH_2_PO_4_, 6 mM glucose, and 20 mM Hepes, pH 7.4). Experiments were performed at room temperature.

The fluorescent dyes were loaded as follows: (i) fura-2 acetoxymethyl ester (Calbiochem), 4 *μ*M, 40 minutes at 37°C; (ii) Sytox orange, 3 *μ*M, kept in the extracellular buffer during the experiments; and (iii) Hoechst 33342, 10 *μ*g/ml, 10 minutes at RT [27, 28].

### 2.6. Cell Treatments

The acute iron overload protocol was performed incubating cells in the presence of 20 *μ*M pyrithione, an iron ionophore, for 2 min before the administration of Fe^2+^ (as FAS, ferrous ammonium sulfate) at the desired concentration for 3 min, followed by several washes with KRH.

In some experiments, cells were pretreated with drugs as follows: (i) Tempol (4-hydroxy-2,2,6,6-tetramethylpiperidine 1-oxyl) and MitoTEMPO ((2-(2,2,6,6-tetramethylpiperidin-1-oxyl-4-ylamino)-2-oxoethyl) triphenylphosphonium chloride monohydrate) (Alexis Biochemicals, Lausen, Switzerland), 100 *μ*M, were added to cells 1 hour before the experiment and kept in the extracellular buffer during the experiment; (ii) L-buthionine sulfoximine (BSO), 300 *μ*M or 1 mM (for cortical neurons and MEFs, respectively), was incubated in the cellular medium overnight.

### 2.7. Measurements of Glutathione

Reduced glutathione (GSH) content was measured at single-cell level by the thiol-reactive fluorescent probe monochlorobimane (mBCl); mBCl turns fluorescent after conjugation with GSH. In the mBCl assay, 50 *μ*M of mBCl was added to KRH buffer at the beginning of the experiments and the kinetics of fluorescent GSH-monochlorobimane adduct formation was analyzed for 40 minutes, until the plateau phase was reached.

### 2.8. Measurements of Cell Viability

Cell viability was quantified by 3-(4,5-dimethylthiazol-2-yl)-2,5-diphenyltetrazolium bromide (MTT) assay. Briefly, MEFs, plated on 24-well plates, were washed once with KRH buffer and exposed to the experimental protocols for 1 h at 37°C. After washing, cells were incubated for 1 h with 0.5 mg/ml MTT in KRH. After removing the extracellular solution, formazan, the MTT metabolic product, was dissolved in DMSO and the absorbance was read at 570 nm.

### 2.9. Cell Transfection

MEFs were transfected with mitochondrial-targeted EYFP (mitoEYFP vector; Clontech, Mountain View, CA, USA), by Lipofectamine 2000 (Thermo Fisher Scientific) according to the manufacturer's instructions. Cells were incubated with the mix of Lipofectamine and DNA for about 3 hours at 37°C and analyzed 24 h after transfection.

### 2.10. Western Blotting

Western blot was performed as described previously [29]. Briefly, samples (20 *μ*g of proteins per lane) were resuspended in denaturating buffer (Tris/HCl 50 mM, EDTA/Na 2.5 mM, SDS 2%, glycerol 5%, dithiothreitol 20 mM, and bromophenol blue 0.01%) and incubated 10 min at 65°C. Proteins were then separated by standard SDS-polyacrylamide gel electrophoresis and electrically transferred onto nitrocellulose membrane. Membranes were blocked with TBS supplemented with 0.1% Tween-20 and 5% skimmed milk powder. Primary antibodies were diluted in TBS-0.1% Tween-20 as follows: rabbit anti-SOD2 (Upstate Biotechnology, Thermo Fisher Scientific) 1 : 1000; rabbit anti-Prx3 (AB Frontier, Seoul, South Korea) 1 : 2000; and mouse anti-catalase (Sigma-Aldrich) 1 : 2000. For loading controls, anti-actin (Sigma-Aldrich) was used. After washing, membranes were incubated with secondary goat anti-rabbit or anti-mouse horseradish peroxidase-conjugated antibodies (Bio-Rad, Hercules, CA, USA) diluted 1 : 2000 in a blocking solution. Protein bands were detected on autoradiographic films by chemiluminescence with the West Pico or West Femto Super Signal substrate (Thermo Fisher Scientific).

### 2.11. Data Analysis

Data are presented as mean ± SEM as specified. Statistical significance was tested using two-way ANOVA or one-way ANOVA followed by Dunnett's (for multiple comparisons against a single reference group) or Bonferroni (for all pair wise comparisons) post hoc tests. Statistical analysis was performed using GraphPad Prism (GraphPad Software, San Diego, CA, USA).

## 3. Results

### 3.1. Effects of *Afg3l2* KO in MEF Cells

Since the defective expression of *Afg3l2* was reported to promote oxidative stress in Purkinje cells [18] and to recapitulate the typical SCA28 mitochondrial dysfunctions in MEFs [4], we investigated the susceptibility of MEF cells obtained from *Afg3l2*-KO mice (KO MEFs) to oxidative conditions.

**Figure 1:**
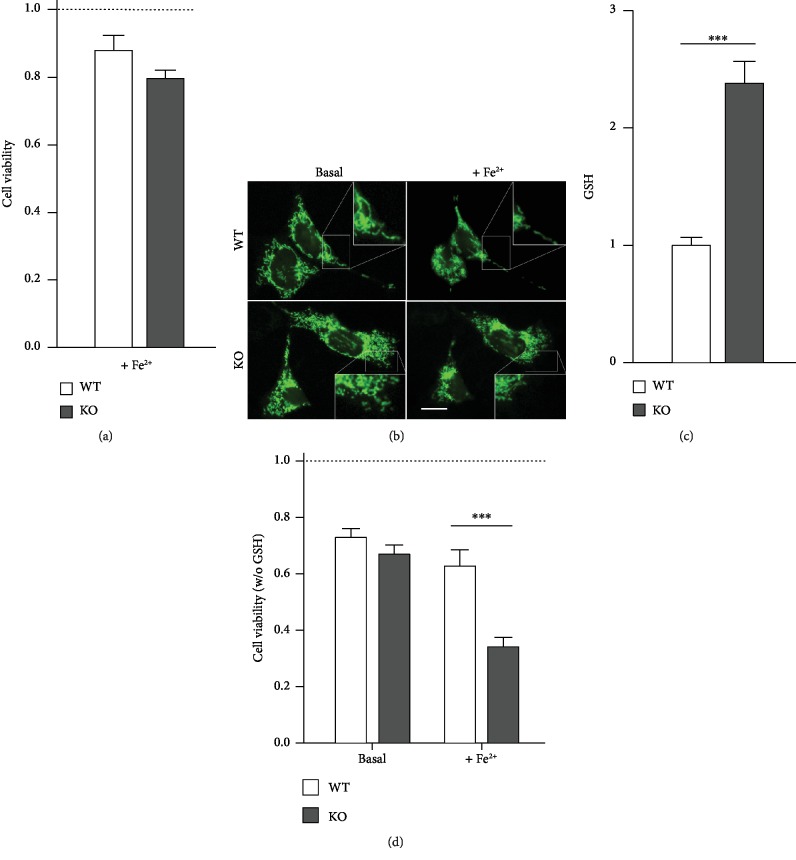
Vulnerability of Afg3l2-KO and WT MEFs to oxidative stress. (a) Acute iron overload (promoted by 100 *μ*M Fe^2+^ administered with the iron ionophore pyrithione, 20 *μ*M) did not significantly alter the cell viability (assessed 90 minutes later by the MTT assay) of either WT or KO MEFs, which both appeared similar to untreated WT cells (dashed line; 7 experiments for each cell lines). (b) WT (top) and KO (bottom) MEFs overexpressing mtEYFP were imaged before (left) and 90 minutes after (right) acute iron overload (performed as in (a)). Insets show a detail of mitochondrial morphology. The acute iron overload does not appear to significantly affect mitochondrial morphology either in WT or KO cells, which appeared similar to their untreated counterpart. Scale bar: 50 *μ*m. (c) Cellular GSH content was estimated at single-cell level by mBCl, a probe that turns fluorescent after conjugation with GSH. Cells were imaged as mBCl was added to the extracellular solution. Bars represent the average, from 4 experiments, of the fold increase (±SEM) of mBCl fluorescence with respect to the basal value. (d) MEFs were depleted of glutathione by o/n treatment with L-buthionine sulfoximine (BSO, 1 mM) before MTT assays; under these conditions, iron overload (performed as in (a)) caused a significant reduction of KO MEF viability, compared to WT counterpart (7 experiments).

MEFs were subjected to protocols of Fe^2+^ overload, in order to favor the Fenton reaction and thus the production of hydroxyl radicals, a highly reactive oxygen species [30]. Acute iron overload was induced by the administration of 100 *μ*M Fe^2+^ in the presence of pyrithione, an iron ionophore that allows a kinetically controlled Fe^2+^ entry [28]. This condition, which is particularly toxic for neuronal cells [28], affected neither the WT nor the KO MEF survival, as assessed by the MTT assay (Figure 1(a)). We further investigated mitochondria to ascertain whether their morphology was affected by the oxidative treatment. As expected from previous reports [4, 16], mitochondrial appearance was already altered in basal conditions: the mitochondrial network was more fragmented in KO MEFs transiently transfected with mtEYFP construct than in WT cells. Acute iron overload did not induce evident morphological alteration in either WT or KO MEFs, even though mitochondria appear redistributed in the perinuclear region (Figure 1(b)). We next investigated possible changes in the basal levels of reduced glutathione (GSH), the main antioxidant molecule in mammalian cells. At steady state, GSH levels were significantly higher in KO than in WT MEFs (mBCl assay; Figure 1(c)). In order to verify the role of GSH in MEF protection, cells were depleted of their glutathione content (overnight treatment with 1 mM buthionine sulfoximine (BSO)), before being exposed to acute iron overload. Under basal condition, GSH depletion similarly reduced cell viability of both WT and KO MEFs; after acute iron overload, a strong toxic effect was observed in KO MEFs but not in WT (Figure 1(d)).

**Figure 2:**
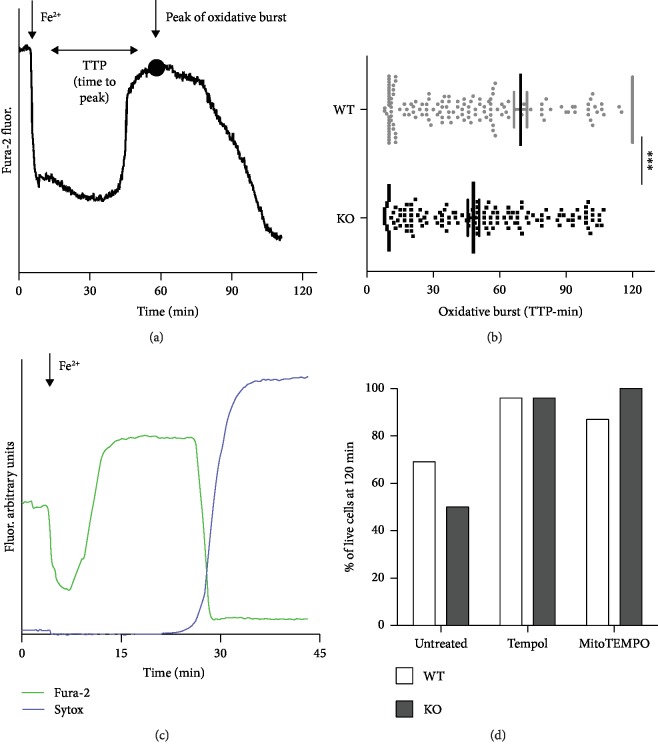
Single-cell analysis in GSH-depleted MEF cells. (a) The acute Fe^2+^ entry was followed kinetically at single-cell level in fura-2-loaded MEFs: pyrithione-mediated Fe^2+^ entry promoted fura-2 fluorescence quenching (excitation at 360 nm, the Ca^2+^-insensitive wavelength) followed, after a variable period of time, by fluorescence recovery due to oxidation of Fe^2+^ to Fe^3+^ likely induced by Fenton reaction. The interval between Fe^2+^ entry and the peak (indicated by a dot in the representative curve) of oxidative burst (TTP: time to peak) occurred significantly early in KO compared to WT MEFs, as described by the graph in (b); ^∗∗∗^*p* < 0.001. (c) The fura-2 dequenching phase can be followed by a loss of fura-2 fluorescence signal (observable also in (a)) as a consequence of an increase in plasmalemma permeability also documented by a concomitant Sytox blue nuclear staining. (d) The percentage of live MEFs (negative for Sytox staining and still loaded with fura-2) at the end of the experiments (120 minutes after acute iron overload) was evaluated, by considering all cells analyzed, under untreated conditions or after pretreatment with 100 *μ*M Tempol and 100 *μ*M MitoTEMPO, cytosolic and mitochondrial ROS scavengers, respectively. Both treatments completely prevented cell death.

Single-cell analysis was used to characterize the kinetics of the responses to acute iron overload after glutathione depletion. Cells were loaded with fura-2, whose fluorescence is selectively quenched by Fe^2+^ but not Fe^3+^ [28], in order to monitor iron oxidation and, indirectly, the Fenton reaction. When the cells were exposed to acute iron overload, the intracellular fura-2 fluorescence at 360 nm was initially quenched by the rapid intracellular Fe^2+^ influx; after a variable period of time, a recovery of the fluorescence signal could be observed (oxidative burst), a sign of the oxidation of Fe^2+^ to Fe^3+^ occurring during the Fenton reaction (Figure 2(a)). The full development of the oxidative burst (i.e., the time to reach the peak (TTP) of fluorescence recovery after iron overload) occurred earlier in KO MEFs than in WT MEFs (with a mean of 48 vs. 70 min; Figure 2(b)). On a wider time window, the average of TTP in WT could be even higher than that indicated. Indeed, the majority of WT MEFs (155 out of 255 cells) was able to maintain iron under a reduced form, without signs of oxidative burst for the entire duration of the experiments (120 min); in this case, the TTP was considered 120 min even though it might have not occurred at all. On the contrary, all KO cells undergo oxidative burst within the same time window, thus indicating a lower reducing capacity. This oxidative burst could be accompanied by alteration of plasma membrane integrity and cell death, a condition revealed by the loss of fura-2 dye and confirmed by Sytox nuclear staining (Figure 2(c)) [28]. Of note, cells were protected when preexposed to ROS scavengers no matter whether acting at the cytosolic (Tempol) or mitochondrial (MitoTEMPO) level, thus indicating the role of oxidative stress in cell death (Figure 2(d)).

Altogether, these results indicate that KO MEFs are constitutively exposed to higher oxidative stress and that they develop higher cellular reducing capacity, mainly attributable to intracellular glutathione potentiation.

### 3.2. Response of *Afg3l2*-KO Cortical Neurons to Oxidative Conditions

Having demonstrated a higher vulnerability of KO MEFs to oxidative insults, we applied the same experimental design to cortical primary neurons obtained by both WT and *Afg3l2*-KO mice (WT and KO neurons, respectively).

**Figure 3:**
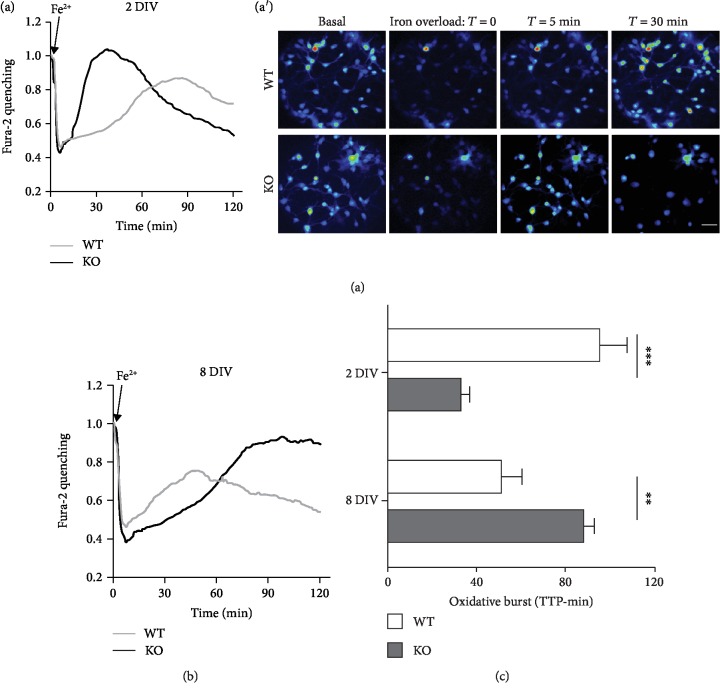
Responses of Afg3l2-KO and WT cortical neurons to oxidative conditions. (a, a′) The graph and the image gallery represent the fura-2 fluorescence of 2 DIV cortical neurons, subjected to protocol of acute iron overload (as described in Figure 1(a), but with 1 *μ*M Fe^2+^ instead of 100 *μ*M). The traces in graphs (a) and (b) correspond to the average of fluorescent signals obtained from all the neurons analyzed per condition (~25 neurons per experiment, from 6-8 independent experiments per condition) and correspond to Fe^2+^ entry (fura-2 fluorescence quenching), followed by iron oxidation (fluorescence recovery) and possibly by neuronal death (fluorescence loss). KO neurons show fluorescence recovery and more massive fluorescence loss, compared to their WT counterpart. Scale bar: 30 *μ*m. (b) At 8 DIV, KO neurons became more resistant to iron overload, with delayed oxidative burst compared to WT neurons and no signs of neuronal death. (c) The comparison of TTP in WT and KO neurons (see (a) and (b)) shows that KO neurons are initially more susceptible to oxidative stress than the WT ones, but they rapidly acquire higher resistance when maintained few days in culture; ^∗∗∗^*p* < 0.001.

Neurons were exposed to the protocol of acute iron overload, in which, however, a more physiological Fe^2+^ concentration (1 *μ*M instead of 100 *μ*M) was administered, to take into account the higher sensitivity of neuronal cells to oxidative insults. For the same reason, neurons were analyzed at the very early stage in culture (2 days in vitro (DIV)), when they are expected to be more resistant to oxidative environment [28]. Despite these attentions to contain the oxidative effects, KO neurons showed a very fast iron oxidation (fura-2 dequenching), an indication of their limited reducing capacity; moreover, shortly after fluorescence recovery, fura-2 signal disappeared from the majority of the cells, a sign of membrane permeabilization preceding cell death. Meanwhile, the age-matched WT neurons appeared more resistant to the iron insult: the increase in the fluorescence signal was typically delayed and did not necessarily lead cells to death during the 2 hours of image acquisition (Figures 3(a) and 3(a′); the two traces in Figure 3(a) represent the time course of averaged fluorescence signals from all neurons analyzed in separate experiments). Neurons were then analyzed after one week in culture, to exacerbate the response to oxidative stress. Indeed, after 8-9 DIV, the oxidative burst was sped up in WT neurons, as already observed in aged hippocampal neurons [28]. Unexpectedly, KO neurons showed a delayed fluorescence recovery and no signs of cell death within the experimental time window (Figure 3(b)). The overall resistance to oxidative stress conditions is analyzed in Figure 3(c), where the peaks of the oxidative bursts in WT and KO neurons are compared at 2 and 8 DIV. The analysis clearly shows that KO neurons are initially more susceptible to oxidative stress (oxidative burst occurring at a mean of 32 and 95 minutes in KO and WT neurons, respectively), but in few days acquire higher resistance than controls (51 and 88 minutes in KO and WT neurons, respectively).

### 3.3. Role of Glutathione in the Protective Mechanisms

**Figure 4:**
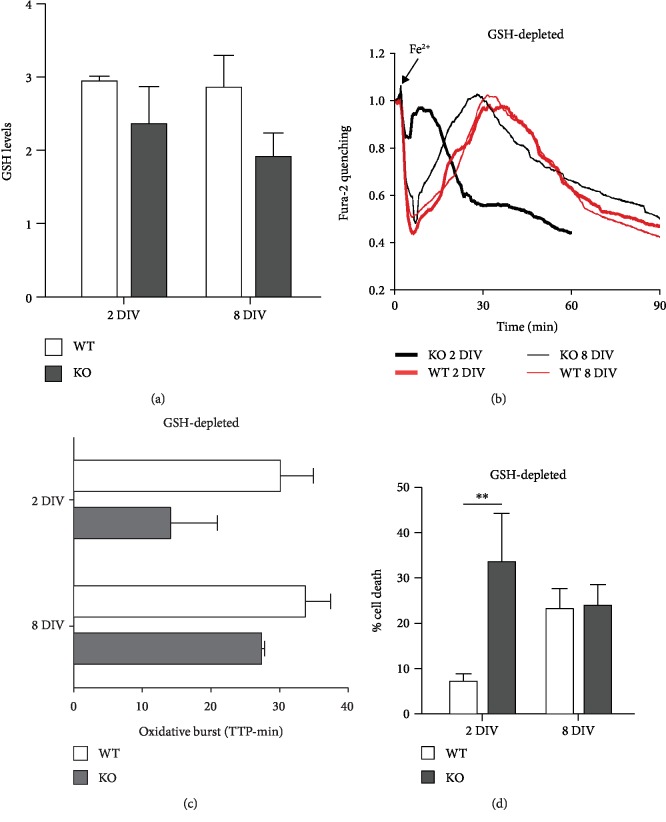
Role of glutathione in the protection of KO cortical neurons from oxidative stress. (a) During aging in culture, GSH content, analyzed by mBCl as described in Figure 1(c), remained at comparable levels in WT neurons, while it showed a decreasing trend in KO neurons, indicating a minor role for GSH in the establishment of protective mechanisms. (b, c) Glutathione depletion (by O/N treatment with BSO, 300 *μ*M) speeds up all cellular processes following Fe^2+^ entry (compared with the 2 graphs in Figures 3(a) and 3(b)), but still, 8 DIV KO neurons are more resistant than the 2 DIV KO ones. This was confirmed by the kinetics of oxidative burst (evaluated as in the previous two figures), therefore indicating no major role of GSH in antioxidant protective mechanism. (d) Under conditions of glutathione depletion (as in (b) and (c)), neuronal death was significantly higher in 2 DIV KO compared to WT neurons, while it was similar in 8 DIV neurons; ^∗∗^*p* < 0.01.

In order to investigate the compensatory mechanism that develops with time in culture and taking into consideration the results obtained in MEF, we assessed the levels of GSH by the mBCl assay. GSH levels were somewhat lower in KO neurons (although not significantly different) at both time points, showing a tendency to further decrease, rather than increase, in the older cells (Figure 4(a)), suggesting that GSH is not the main responsible for the observed protective mechanism. The role of this antioxidant molecule was further investigated in a second set of experiments, in which neurons were virtually depleted of GSH (overnight incubation with 300 *μ*Μ BSO) before being subjected to iron-overload protocol and single-cell analysis. The four traces in Figure 4(b), which represent the mean of all neurons analyzed in each condition, show a much faster fluorescence recovery and fluorescence loss, compared to the untreated counterpart (Figures 3(a) and 3(b)), suggesting lower cellular reducing capability and higher neuronal death. The bar graph in Figure 4(c) showed that GSH depletion speeds up all kinetics, thus making the TTP values not significantly different. Nonetheless, even without GSH, the oxidative burst appeared still delayed in 8 DIV KO neurons (a TTP mean of 14 versus 27 minutes in neurons of 2 and 8 DIV, respectively). These data were further confirmed by a quantitative analysis of the neuronal death, performed by high-throughput microscopy (Array Scan XTI platform, Thermo Fisher Scientific). As soon as 30 minutes after Fe^2+^ administration, the 2 DIV KO neurons showed an elevated percentage of death, significantly higher than the WT counterpart. At 8 DIV, the toxicity increased in WT and decreased in KO neurons reaching comparable values (Figure 4(d)).

Overall, even in conditions of glutathione depletion, the KO neurons appeared more resistant to iron-mediated oxidative stress at 8 DIV than at 2 DIV, thus suggesting that other protective mechanisms play a major role.

### 3.4. Role of Mitochondrial SOD2 and Prx3 Enzymes

**Figure 5:**
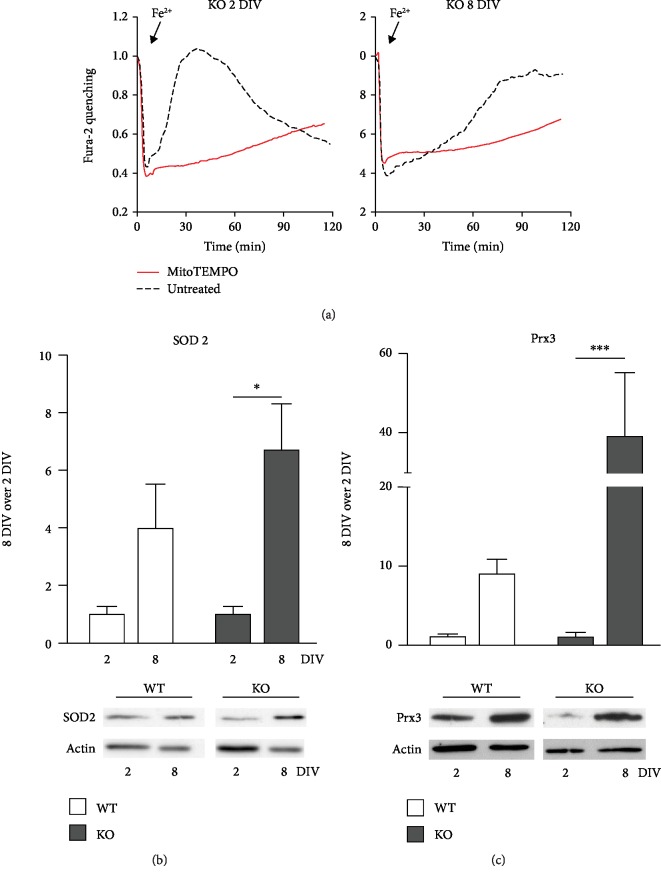
Role of mitochondrial SOD2 and Prx3 enzymes. (a) The pretreatment of KO neurons with MitoTEMPO (red traces, averaged from all neurons of three representative experiments), a mitochondrial-targeted superoxide scavenger, prevents the oxidative burst and the loss of fura-2 dye that, instead, occurs in black dotted traces (untreated cells, as in Figures 3(a) and 3(b)) in both 2 and 8 DIV neurons. (b, c) Representative lanes (from a single western blot) and quantification (from 3 separate experiments, with all values first normalized on actin levels) reveal an increase in both SOD2 and Prx3 expression from 2 DIV to 8 DIV; this raise was much more remarkable in KO neurons than in WT. ^∗^*p* < 0.05; ^∗∗∗^*p* < 0.001.

Altogether, our results ruled out a prominent role for GSH in the compensatory and protective mechanisms that develop in cortical neurons during aging in culture. Accordingly, we investigated other protective pathways, paying specific attention to the role played by mitochondria for two main reasons: (i) AFG3L2 is a mitochondrial protein and its deficiency affects mainly these organelles; (ii) in hippocampal neurons, the iron-mediated oxidative stress causes a mitochondrial impairment and fragmentation that can be prevented only by MitoTEMPO, a mitochondrial-targeted superoxide scavenger [28]. Indeed, when cortical KO neurons were pretreated (1 h) with MitoTEMPO, before exposure to acute iron overload, the oxidative burst was abolished and the neuronal death was completely prevented at both 2 and 8 DIV, as shown in the two graphs of Figure 5(a), where the responses of treated (red traces, averaged from all cells analyzed in 3 separate experiments) and untreated (black dotted traces, already shown in Figures 3(a) and 3(b)) cells were superimposed. Similar results were obtained with 8 DIV WT neurons (not shown). Since these data provide a direct evidence of an involvement of mitochondria, we investigated the possible contribution of different antioxidant enzymes residing in these organelles. Manganese superoxide dismutase (MnSOD or SOD2) plays a crucial role in the scavenging of the superoxide anion, and its expression can be controlled in different physiological and pathological conditions. The analysis of SOD2 protein expression in our cultured neurons revealed an increase with time in culture (from 2 to 8 DIV), which was much more evident in KO neurons than in WT (Figure 5(b)).

However, an increased competence of neurons in dismutation of superoxide anion implies a more efficient production of hydrogen peroxide that in turn has to be detoxified. The conversion of hydrogen peroxide into water can be catalyzed by 3 classes of redox enzymes: glutaredoxins, catalase, and peroxiredoxins. The first group is GSH-dependent; therefore, based on our previous experiments, it is not expected to be primarily involved in the protective mechanisms that develop in culture. Catalase is expressed on peroxisomes and, from our biochemical data, does not increase with the time in culture (supplementary [Supplementary-material supplementary-material-1]). We therefore considered Prx3 that is mainly expressed in the mitochondrial matrix and is responsible for 90% of hydrogen peroxide detoxification [31]. The Prx3 expression was very low at 2 DIV (western blot in Figure 5(c)), a condition that can explain their high susceptibility to oxidative stress but showed a remarkable increase in 8 DIV KO neurons; although the increase was significant also in WT neurons (~10 times), it was even more impressive in KO neurons (~40 times; Figure 5(c)).

## 4. Discussion

Several neurodegenerative disorders are characterized by a selective neuronal vulnerability, meaning that subpopulations of neurons are more prone to death in response to a common pathological condition [32]. This selective toxicity may be caused by a higher expression of the altered gene, by specific features that make neurons intrinsically more vulnerable to insults [33], or by their lower competence to develop compensatory mechanisms against the neurotoxic conditions. In neurodegenerative disorders primarily involving mitochondrial alterations (such as SCA28, Friedreich's ataxia [34], and HSP-SPG7 [1]), oxidative stress is expected to contribute to the neurotoxic process.

In this study, we exposed two cell types, MEF cells and cortical neurons from Afg3l2-KO mice to oxidative insults by acute iron overload [28] in order to challenge the cellular redox homeostasis and also because the accumulation of intracellular iron reproduces, within the experimental time window, a condition typical of several neurodegenerative disorders as well as of normal brain during aging [35–37].

The evidence that iron overload does not affect KO MEFs more than WT indicates that mutants acquire higher resistance to oxidative stress to compensate for the mitochondrial defects. The significant increase in GSH levels found in KO cells can, at least partially, account for this enhanced antioxidant competence; indeed, glutathione depletion reduces the ability of KO MEFs to maintain the physiological redox state, therefore increasing the susceptibility to the effects of iron overload compared to the WT counterpart. The higher GSH production in mutants could be mediated by an increase in cellular cystine levels, which are known to be controlled by oxidative stress. Indeed, the cystine/glutamate antiporter system X_c_- is induced by the NF-E2-related factor 2 (Nrf2) transcription factor, via the antioxidant response element (ARE) [38]. Moreover, Nrf2, together with other transcription factors such as AP-1 and NF*κ*B, contributes to regulate the expression of glutamate cysteine ligase and glutathione synthetase, the two main enzymes involved in glutathione synthesis [39].

Whatever the mechanism, the evidence of a compensatory antioxidant mechanism at the steady state confirms the presence of higher basal levels of oxidative stress in *Afg3l2*-deficient MEFs and indicates the cellular ability of maintaining this protective competence over time. The capability of the cells to become resistant to oxidative challenge is particularly important for neurons, which intrinsically produce more ROS and are more susceptible to ROS production, given their lower level of antioxidant defenses. Indeed, our data showed that *Afg3l2*-KO neurons 2 days after plating were more sensitive than their WT counterpart to the oxidative conditions. On the other hand, as mentioned before, KO cortical neurons developed, very early in culture, mechanisms able not only to maintain under control oxidative stress consequent to *Afg3l2* deficiency but also to counteract the increased susceptibility to oxidative conditions observed in the aged neurons [28]. Indeed, while the WT older neurons decrease their reducing capability, the 8 DIV KO neurons become more resistant to oxidative damage through mechanisms that are different from those established in MEFs. GSH appears to be important for KO MEFs but less for cortical KO neurons, possibly because of its very low level in neuronal cells [28, 40]. In our *Afg3l2*-KO neuronal model, antioxidant defenses can be primarily attributed to the induction of the mitochondrial enzymes SOD2 and Prx3. SOD2 detoxifies the superoxide by converting it into hydrogen peroxide [41], in physiological conditions as well as in some neurodegenerative diseases, where it can contribute to prevent neurodegeneration. However, high levels of SOD2 may have detrimental effects on neuronal lifespan, if the increased hydrogen peroxide is not efficiently detoxified by glutaredoxins, catalase, or peroxiredoxins [42]. This was certainly not the case in older *Afg3l2*-KO neurons, where Prx3 protein levels were massively increased. The role played by Prx3 in scavenging mitochondrial hydrogen peroxide has recently attracted considerable attention. In particular, data in the literature directly correlate Prx3 expression level with a neuroprotective role under oxidative conditions [43–45]. For instance, an upregulation of Prx3 was observed in hippocampal pyramidal neurons as soon as one day after cerebral ischemia/reperfusion and was reported to remain high for several days, contributing to protect neurons from ischemic damage [46]. Moreover, neuronal death caused by excitotoxic hippocampal injury was prevented by *in vivo* Prx3 overexpression [47]. On the other hand, when Prx3 expression is downregulated, higher sensitivity to oxidative stress is observed; this is reported in Fanconi anemia [48], in brains of Alzheimer's and Down syndrome patients [49], and in the motor neurons of amyotrophic lateral sclerosis patients [50]. Prx3 is regulated at the transcriptional level by the FOXO (Forkhead box, class O) subfamily of Forkhead transcription factors, which are regulators of antioxidant cellular response and whose activity is, in turn, modulated by ROS levels [51]. Our results suggest that 8 DIV cultured *Afg3l2*-KO neurons reach an oxidative stress level that, instead of making them more susceptible to external oxidative insults, triggers a protective program in which massive upregulation of Prx3 confers a resistance to stress even higher than that of WT neurons.

We hypothesize that this emergency escape of ROS challenge is neuronal-specific and contributes to degeneration of selective populations of neurons that failed to activate the Prx3-associated detoxifying pathway. In this context, it will be important to evaluate whether the PCs from SCA28 models are as efficient as the cortical neurons in developing this compensatory antioxidant pathway or, conversely, if they cannot prevent or compensate ROS production. In this case, the oxidative stress induced by mitochondrial impairment might contribute to alter mitochondrial trafficking, as reported by Kondadi and coworkers [52]. On the other hand, ROS elevation can participate to calcium dysregulation [53] or directly contribute to the neurodegenerative process affecting PCs in SCA28.

Finally, it might be also interesting to assess whether the Prx3-dependent protective pathway is also activated in other neurodegenerative diseases involving mitochondria and oxidative stress, such as Friedreich's ataxia [54, 55], which might represent a good model to test this hypothesis.

Overall, our data support the antioxidant role of Prx3 in neuronal cells *in vitro* and open new perspective for the study of its involvement also in *in vivo* models, also in view of its recognized role in neuronal disorders associated to oxidative conditions [56].

## Data Availability

The data used to support the findings of this study are available from the corresponding author upon request.

## Supplementary Materials

Supplementary Materials Supplementary Figure S1: Representative western blot and quantification (neurons obtained form 5 KO and 6 WT mice) do not reveal an increase in catalase expression between 2 and 8 DIV.
