# Supplementary material for: Upregulation of Peroxiredoxin 3 Protects Afg3l2-KO Cortical Neurons In Vitro from Oxidative Stress: A Paradigm for Neuronal Cell Survival under Neurodegenerative Conditions
Source: Oxid Med Cell Longev. 2019 Oct 31;2019:4721950. doi: 10.1155/2019/4721950 (PMC6875171; doi:10.1155/2019/4721950)
Supplement: Supplementary Materials — Supplementary Figure S1: Representative western blot and quantification (neurons obtained form 5 KO and 6 WT mice) do not reveal an increase in catalase expression between 2 and 8 DIV. [file 4721950.f1.pdf]

S1

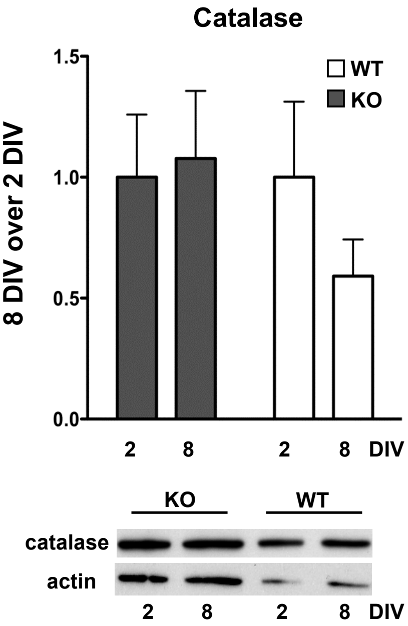

Representative western blot and quantification (neurons obtained form 5 KO and 6 WT mice) do not reveal an increase in catalase expression between 2 and 8 DIV.
